# Therapeutic Effect of Nanogel-Based Delivery of Soluble FGFR2 with S252W Mutation on Craniosynostosis

**DOI:** 10.1371/journal.pone.0101693

**Published:** 2014-07-08

**Authors:** Masako Yokota, Yukiho Kobayashi, Jumpei Morita, Hiroyuki Suzuki, Yoshihide Hashimoto, Yoshihiro Sasaki, Kazunari Akiyoshi, Keiji Moriyama

**Affiliations:** 1 Maxillofacial Orthognathics, Department of Maxillofacial Reconstruction and Function, Division of Maxillofacial/Neck Reconstruction, Graduate School of Medical and Dental Sciences, Tokyo Medical and Dental University, Tokyo, Japan; 2 Hard Tissue Genome Research Center, Tokyo Medical and Dental University, Tokyo, Japan; 3 Department of Polymer Chemistry, Graduate School of Engineering, Kyoto University, Kyoto, Japan; 4 ERATO, Japan Science and Technology Agency, Tokyo, Japan; Inserm U606 and University Paris Diderot, France

## Abstract

Apert syndrome is an autosomal dominantly inherited disorder caused by missense mutations in fibroblast growth factor receptor 2 (FGFR2). Surgical procedures are frequently required to reduce morphological and functional defects in patients with Apert syndrome; therefore, the development of noninvasive procedures to treat Apert syndrome is critical. Here we aimed to clarify the etiological mechanisms of craniosynostosis in mouse models of Apert syndrome and verify the effects of purified soluble FGFR2 harboring the S252W mutation (sFGFR2IIIc^S252W^) on calvarial sutures in Apert syndrome mice *in vitro*. We observed increased expression of Fgf10, Esrp1, and *Fgfr2IIIb*, which are indispensable for epidermal development, in coronal sutures in Apert syndrome mice. Purified sFGFR2IIIc^S252W^ exhibited binding affinity for fibroblast growth factor (Fgf) 2 but also formed heterodimers with FGFR2IIIc, FGFR2IIIc^S252W^, and FGFR2IIIb^S252W^. Administration of sFGFR2IIIc^S252W^ also inhibited Fgf2-dependent proliferation, phosphorylation of intracellular signaling molecules, and mineralization of *FGFR2^S252W^*-overexpressing MC3T3-E1 osteoblasts. sFGFR2IIIc^S252W^ complexed with nanogels maintained the patency of coronal sutures, whereas synostosis was observed where the nanogel without sFGFR2^S252W^ was applied. Thus, based on our current data, we suggest that increased Fgf10 and *Fgfr2IIIb* expression may induce the onset of craniosynostosis in patients with Apert syndrome and that the appropriate delivery of purified sFGFR2IIIc^S252W^ could be effective for treating this disorder.

## Introduction

Genetic mutations in fibroblast growth factor receptors (*FGFR*) 1–3 cause several types of syndromic craniosynostosis, including Apert syndrome (AS; OMIM #101200) [1], [2], [3], [4], [5]. Most cases of AS are caused by either one of the two missense mutations in *FGFR2* exon 7, i.e., C934G or C937G leading to the amino acid substitutions S252W or P253R, respectively [5]. These mutations are known to enhance ligand-dependent activation of FGFR2 by reducing the dissociation rate between ligands and FGFR2; loss of ligand specificity in turn causes aberrant binding of FGFR2IIIc, a mesenchymal splicing isoform, to FGF7 or FGF10 [6], thereby inducing enhanced differentiation of osteoblasts [7]. We previously reported that a soluble form of FGFR2IIIc with the S252W mutation (sFGFR2IIIc^S252W^), which is truncated at the extracellular domain, inhibits the aberrant mineralization of MG63 cells overexpressing full-length FGFR2IIIc^S252W^ [8]. We also demonstrated that calvarial osteoblasts isolated from transgenic mice overexpressing sFGFR2IIIc^S252W^ exhibit reduced tyrosine phosphorylation of signaling molecules in the mitogen-activated protein kinase (MAPK) pathway, lower expression of osteoblastic marker genes, and impaired mineralization [9]. In addition, we developed a mouse model of AS expressing the sFGFR2IIIc^S252W^ protein and demonstrated that these mice exhibit partially rescued coronal sutures *in vivo* [10]. These results indicated that sFGFR2IIIc^S252W^ may act as an inhibitor of phenotypic expression in AS osteoblasts.

Cholesteryl-bearing pullulan (CHP), which is composed of hydrophilic polysaccharides partially modified with hydrophobic cholesteryl groups, self-assembles in water and forms stable nanogels with a diameter of 30 nm [11], [12]. The CHP nanogel has two unique characteristics as follows: 1) a high loading capacity for bioactive molecules inside their nano spaces with polymer networks and 2) a chaperone-like activity which enables the delivery of a variety of molecules to the targeted sites. To date, the CHP nanogel has been utilized as a drug delivery system for multiple purposes, such as cancer treatment [13], [14], [15], [16], nasal vaccines [17], and cytokine therapy [18]. More recently, the nanogel-crosslinked hydrogels were also developed by Michael addition of acryloyl-bearing CHP (CHPOA) to PEG containing four branched terminal thiol groups (PEGSH). These macrogels have been utilized as a scaffold material for controlled drug release in regenerative medicine [19], [20].

We hypothesize that sFGFR2^S252W^ may act as a suppressor for hyperdifferentiation of osteoblasts in the coronal sutures of AS mice. The aim of this study was to elucidate the pathogenic mechanisms of craniosynostosis in AS and verify the therapeutic applicability of purified sFGFR2IIIc^S252W^ delivered via a polysaccharide nanogel as a protein carrier in Apert calvarial sutures.

## Materials and Methods

### Animals

All animal experiments were performed in accordance with the protocols approved by the Institutional Animal Care and Use Committee of Tokyo Medical and Dental University (Permission number: 0110009B). All samples were extracted from mouse embryos at embryonic day 15.5 (E15.5). All efforts were made to minimize animal suffering. AS mice (*Fgfr2^+/S252W^* mice) were generated by mating male *Fgfr2^+/Neo–S252W^* mice and female +/Ella-Cre mice. Polymerase chain reaction (PCR) genotyping of progeny mice was performed using tail genomic DNA isolated with a DNeasy Blood and Tissue Kit (Qiagen, Crawley, UK), KOD Plus Polymerase (Toyobo, Osaka, Japan). The following specific primers were used for the PCR: 5′-TAGGTAGTCCATAACTCGG-3′ and 5′-TTGATCCACTGGATGTGGGGC-3′. While single fragments were amplified from the genomic DNA of *Fgfr2^+/Neo–S252W^* mice (457 bp) and +/EIIa-Cre mice (393 bp), both 457-bp and 393-bp fragments were amplified from that of AS *Fgfr2^+/S252W^* mice. Littermates were used as controls.

### RNA preparation, reverse transcription (RT)-PCR, and real-time PCR

Embryonic calvarial coronal sutures (E15.5) were dissected from AS mice and control littermates under a stereoscopic microscope. Both sutures from each calvaria were collected and combined. To isolate total RNA, tissues were lysed in Isogen (Nippon Gene, Toyama, Japan) according to the manufacturer's instructions. Reverse transcription was performed using a PrimeScript First Strand cDNA Synthesis Kit (Takara, Shiga, Japan). Real-time PCR was performed using TaqMan gene expression assays on a 7300 Fast Real-Time PCR System (Applied Biosystems, Carlsbad, CA, USA), according to the manufacturer's instructions. Primers and TaqMan probes were designed for the following genes: *Runx2* (encoding mouse *Runx2*; Mm00501580_m1) and *Opn* (encoding mouse *Osteopontin*; Mm01611440_mH). Relative expression levels were calculated using the level of *β-actin* mRNA in each sample as a reference. The expression levels of *Fgfr2IIIb*, *IIIc*, *Fgf10*, and *Esrp1* mRNA were analyzed by real-time PCR using SYBR Green PCR Master Mix (Applied Biosystems) with the following primer pairs: *Fgfr2IIIb*, 5′-AGCTCCAATGCAGAAGTGCTGGC-3′ (forward) and 5′-TGTTTGGGCAGGACAGTGAGCC-3′ (reverse); *Fgfr2IIIc*, 5′-CCACGGACAAAGAGATTGAGGT-3′ (forward) and 5′-TGTCAACCATGCAGAGTGAAAG-3′ (reverse); *Fgf10*, 5′-GGCCACCAACTGCTCTTCTT-3′ (forward) and 5′-TCGTCATGGGGAGGAAGTGA-3′ (reverse); *Esrp1*, 5′-GCCCTCCGACAGTTTAACCA-3′ (forward) and 5′-TGCCACTTTCAAACTTGTAGTTTAC-3′ (reverse); *β-actin*, 5′-TGCGTGACATCAAAGAGAAG-3′ (forward) and 5′-GATGCCACAGGATTCCATA-3′ (reverse). All samples were assayed in triplicate according to the manufacturer's recommendations. Data were analyzed using the comparative Ct method with normalization to the housekeeping gene *β-actin*.

### Analysis of intracellular signaling

Embryonic calvarial coronal sutures (E15.5) were lysed in RIPA buffer supplemented with protease inhibitors (Complete Mini; Roche Diagnostics, Indianapolis, IN, USA). Protein extracts (10 µg each) from paired sutures from embryos were separated by sodium dodecyl sulfate-polyacrylamide gel electrophoresis (SDS-PAGE) and transferred to nitrocellulose membranes. Membranes were then incubated with specific primary antibodies for 12 h at 4°C, followed by incubation with horseradish peroxide (HRP)-conjugated secondary antibodies. Protein bands were developed using Amersham ECL Prime Western Blotting Detection Reagent (GE Healthcare, UK) and visualized using an LAS 4000 system (Fujifilm, Tokyo, Japan). Primary antibodies, including anti-extracellular signal-regulated kinase (ERK, 9102), anti-phospho (p)-ERK (9101), anti-MAPK kinase (MEK, 9122), anti-p-MEK (91221), anti-stress-activated protein kinase (SAPK)/c-Jun N-terminal kinase (JNK, 9252), anti-p-SAPK/JNK (9251), anti-p38 (9212), anti-p-p38 (9211), anti-Akt (9272), anti-p-Akt (9271), and anti-Bax (2772), were obtained from Cell Signaling Technology (Danvers, MA, USA). Anti-Esrp1/2 monoclonal antibodies (201-301-C31S) and anti-β-actin monoclonal antibodies (A1978) were purchased from Rockland (Gilbertsville, PA, USA) and Sigma-Aldrich (Poole, UK), respectively.

### Purification of sFGFR2IIIc and sFGFR2IIIc^S252W^

The culture medium from Cos-7 cells stably expressing sFGFR2IIIc-FLAG or sFGFR2IIIc^S252W^-FLAG [8] was concentrated using an Amicon Ultra-4 Centrifugal Filter Unit (Millipore, Billerica, MA, USA). Both sFGFR2IIIc and sFGFR2IIIc^S252W^ proteins were purified using the FLAG M Purification Kit (Sigma-Aldrich), and purification was subsequently verified by western blot analysis with an anti-FLAG antibody (F3165; Sigma-Aldrich).

### Pull-down Assay

First, we produced His-tagged Fgf2 protein as follows. Murine primary lung cells were obtained from E15.5 wild-type mice, and RNA was extracted using Isogen (Nippon Gene). RT-PCR was performed using the following primer pair: *Fgf2*, 5′-GCTGCCAGCGGCATCACCTC-3′ and 5′-GCTCTTAGCAGACATTGGAA-3′. PCR products of the *Fgf2* (459-bp) amplicon were extracted and purified using a Gel Extraction Kit (Qiagen), followed by ligation into the TOPO II vector (Invitrogen, Carlsbad, CA, USA) using a Quick Ligation Kit (New England Biolabs, Ipswich, MA, USA). Using generated plasmids as templates, cDNA samples of *Fgf2* were subjected to PCR amplification with the following specific primer pair: *Fgf2*, 5′-GACTAGTGCCATGGCTGCCAGCGGCATCA-3′ and 5′-GGAATTCGCTCTTAGCAGACATTGGAA-3′ (with SpeI/EcoRI sites). The amplified product digested with SpeI/EcoRI was subcloned in frame into the pTracer-EF/V5-His expression vector (Invitrogen). After the sequences of the resulting expression vectors were confirmed by sequencing, these vectors were used to express Fgf2-His proteins. Purification of Fgf2-His proteins was then performed using the MagneHis Protein Purification System (Promega, Southampton, UK). With purified sFGFR2IIIc and sFGFR2IIIc^S252W^ as bait proteins and Fgf2-His as a prey protein, pull-down assays were carried out using a Pull-down PolyHis Protein-Protein Interaction Kit (Thermo Scientific, Waltham, MA, USA), according to the manufacturer's instructions.

### Immunoprecipitation and western blot analysis

After Cos-7 cells reached 80% confluence in 10-cm culture dishes, cells were transfected with 4 µg of the following plasmids using Attractene Transfection Reagent (Qiagen): (1) FLAG-MOCK; (2) FGFR2IIIb^S252W^-Zeo(-) (full-length FGFR2IIIb with the S252W mutation subcloned into pcDNA™3.1/Zeo(-) from Invitrogen); (3) FGFR2IIIc^S252W^-FLAG and sFGFR2IIIc^S252W^-FLAG; (4) FGFR2IIIc^S252W^-FLAG and sFGFR2IIIc^S252W^-FLAG; (5) FLAG-MOCK and sFGFR2IIIc^S252W^-FLAG; or (6) FGFR2IIIb^S252W^-Zeo(-) and sFGFR2IIIc^S252W^-FLAG. After 2 h, the culture medium was changed to fresh minimum essential medium (MEM)-α containing fetal bovine serum (FBS). After 24 h, cells were lysed in 1 mL RIPA buffer (50 mM Tris-HCl, 150 mM NaCl, 1% Nonidet P-40 [Nakarai Chemicals, Ltd., Kyoto, Japan], and 0.5% sodium deoxycholate [Wako, Osaka, Japan]) containing protease inhibitors (Complete Mini; Roche), followed by ultrasonication for 5 s. For immunoprecipitation, protein extracts (55 µg) were incubated with anti-FGFR2 polyclonal antibodies (#PA1-24763; Thermo Scientific) overnight at 4°C and then with 30 µL of TrueBlot Anti-Rabbit Ig IP Beads (Rockland) for 2 h at 4°C. IP beads-antibody-antigen complexes were washed five times with IP buffer, mixed with 100 µL sample buffer (4% SDS, 20% glycerol, 10% 2-mercaptoethanol, 0.125 M Tris-HCl, pH 6.8; Sigma), and then boiled. For western blot analysis, supernatant samples were separated by SDS-PAGE and electrotransferred to polyvinylidene difluoride membranes (Bio-Rad, Hercules, CA, USA), followed by incubation with polyclonal anti-FGFR2 (Thermo Scientific) or anti-FLAG (Sigma) antibodies. As the secondary antibody, Rabbit TrueBlot: Anti-Rabbit IgG HRP (Rockland) was used in western blot analysis to avoid detecting IgG bands. Subsequently, proteins were visualized using an ECL Advance Kit (Amersham Biosciences, Piscataway, NJ, USA), and radiographic films were photographed with an LAS4000 system (Fujifilm).

### Establishment of MC3T3-E1 cells stably expressing FGFR2IIIc^S252W^-FLAG

An MC3T3-E1 cell clone was maintained in MEM-α supplemented with L-glutamine, phenol red (Wako), 10% FBS, and antibiotics (100 IU/mL penicillin and 100 IU/mL streptomycin; Invitrogen) at 37°C in an atmosphere containing 5% CO_2_. MC3T3-E1 cells grown in 100-mm culture dishes until 80% confluence were transfected with 10 µg of the FGFR2IIIc^S252W^-FLAG-expressing plasmid using Attractene Transfection Reagent. Twenty-four hours post-transfection, cells were trypsinized, plated on 100-mm culture dishes, and maintained in MEM-α containing 10% FBS and 400 µg/mL G418 (Geneticin; Sigma) for 2 weeks. Resulting colonies were isolated using cloning cylinders and transferred to 24-well plates. A clone of MC3T3-E1 cells stably expressing FGFR2IIIc^S252W^-FLAG (MC3T3-Ap) was established for further analyses.

### 3-(4,5-Dimethylthiazol-2-yl)-2,5-diphenyltetrazolium bromide (MTT) assay

MC3T3-E1 and MC3T3-Ap cells were cultured at 37°C in an atmosphere containing 5% CO_2_ in the presence of U0126 (a MEK inhibitor, 20 µM; Cell Signaling Technology), SB203580 (a p38 inhibitor, 20 µM; Calbiochem, San Diego, CA, USA), sFGFR2IIIc, or sFGFR2IIIc^S252W^ (60 ng/mL) with FGF2 (25 ng/mL). After 48 h, cell proliferation was analyzed by MTT assay. Briefly, 10 µL of MTT solution (5 mg/mL in phosphate-buffered saline [PBS], pH 7.6) was added to each well, and plates were incubated for 4 h. Reactions were terminated by adding 100 µL of dimethyl sulfoxide to each well. The absorbance was read at 570 nm (with 630 nm as the reference) using a multiwell spectrophotometer (Bio-Rad). Each sample included 16 replicates, which were used for statistical analysis.

### Effects of sFGFR2IIIc and sFGFR2IIIc^S252W^ on the phosphorylation of intracellular signaling molecules

MC3T3-Ap cells were cultured in the presence of FGF2 (25 ng/mL), U0126 (20 µM), SB203580 (20 µM), sFGFR2IIIc, or sFGFR2IIIc^S252W^ (60 ng/mL) for 4 h at 37°C in an atmosphere containing 5% CO_2_, as described before. Cells were then lysed in RIPA buffer and sonicated for 5 s. Proteins (10 µg) were loaded onto 10% SDS-PAGE gels and transferred to polyvinylidene difluoride membranes (Amersham Biosciences), followed by incubation with primary antibodies specific for various intracellular signaling molecules as described above.

### Matrix mineralization

Matrix mineralization was induced *in vitro* using MEM-α containing 10% FBS, ascorbic acid (50 µg/mL), dexamethasone (1×10^−8^ M), and β-glycerophosphate (10 mM) in the presence of U0126 (20 µM), SB203580 (20 µM), or sFGFR2IIIc^S252W^ (60 ng/mL) for the indicated culture periods (1–3 weeks). These concentrations were based on those used in previous reports [9]. The mineralized matrix was then stained with Alizarin Red S (AR-S, pH 4.2; Wako) as follows. Briefly, at each time point, cells were washed twice with cold PBS and fixed with cold 70% ethanol for 60 min at 4°C. Cells were then incubated for 20 min with 40 mM AR-S with shaking. To minimize any nonspecific staining, cells were rinsed 5 times with deionized water and once with PBS for 20 min. Finally, AR-S staining of extracellular matrix mineralization was photographed.

### Preparation of nanogel-crosslinked hydrogels complexed with sFGFR2IIIc^S252W^

CHPOA was synthesized by reacting CHP (MW, 1.0×10^5^ Da; 1.2 cholesteryl groups per 100 glucose units) with 2-(acryloyloxy) ethyl isocyanate. The degree of substitution, as determined by ^1^H-nuclear magnetic resonance (NMR), was 10.5 per 100 anhydrous glucoside units. Nanogel-crosslinked hydrogels were prepared by Michael addition of acryloyl groups in CHPOA and terminal thiol groups in PEGSH (MW, 1.0×10^4^; NOF Corporation) as follows: Rhodamine-labeled CHPOA nanogels with or without sFGFR2IIIc^S252W^ were prepared separately. CHPOA nanogels were mixed with PEGSH with a 1∶8 molar ratio of acryloyl groups to thiol groups. Five-microliter aliquots of CHPOA/PEGSH mixtures were then placed between two glass slides coated with Parafilm and incubated for 2 h at 37°C in a humidified atmosphere to obtain disc-shaped nanogel-crosslinked hydrogels incorporating FGFR2^S252W^ (thickness and diameter, approximately 0.5 mm and 2.5 mm, respectively; sFGFR2IIIc^S252W^, 60 ng).

### Calvarial tissue culture

Calvarial bones (E15.5) were dissected from underlying mouse brains, and the skin was peeled off. Tails were used for genotyping as described above. The calvaria from each embryo was bisected in two along the midline of interfrontal and sagittal sutures using ophthalmic instruments under a stereoscopic microscope, taking extreme care not to damage the coronal suture. Bones were then placed in two separate dishes. Explants were cultured using a Trowell-type organ culture system (the grid method) [21]. Briefly, the explants were placed on filters (pore size, 0.1 µm; Whatman, Maidstone, UK) supported by a metal mesh, with the brain side oriented down and the skin side oriented up. The explants were then cultured in 10% FBS-DMEM containing 100 µg/mL ascorbic acid at 37°C in an atmosphere containing 5% CO_2_ under humidified conditions for 4 days, in the presence of either nanogel-crosslinked hydrogels complexed with sFGFR2IIIc^S252W^ or vehicle nanogels placed across the coronal suture (Figure S1). 5-Bromo-2-deoxyuridine (BrdU) was added to the media at a final concentration of 10 µM for 3 h prior to fixation. S-phase cells were then detected using an *In Situ* Cell Proliferation Kit (Roche), according to the manufacturer's instructions. At least four explants from AS mice were sectioned, and at least four sections from each explant were used to quantify the number of BrdU-positive cells.

### Histology

Tissues were fixed in 4% paraformaldehyde in PBS for 24 h at 4°C and were then dehydrated in a graded series of ethanol, embedded in paraffin, and sectioned at 7-µm intervals. After the sections were dewaxed and rehydrated, they were stained with hematoxylin and eosin (Wako). To detect whether sFGFR2IIIc^S252W^ was applied, immunohistochemical staining was performed using polyclonal anti-FLAG (primary) and anti-rabbit IgG HRP-conjugated (secondary) antibodies. Immunoreactivity was subsequently visualized using a VECTASTAIN ABC Kit (Vector Laboratories, Peterborough, UK) according to the manufacturer's instructions. Sections were counterstained with Nuclear Fast Red (Vector Laboratories). BrdU incorporation was also examined as described above. For immunodetection of incorporated BrdU, cells were incubated with fluorescein isothiocyanate-conjugated anti-BrdU monoclonal antibodies for 45 min at 37°C. Cell nuclei were stained with 5 mg/mL 4′,6-diamidino-2-phenylindole (DAPI; Sigma) in PBS prior to mounting slides. Fluorescence was visualized using a fluorescence microscope (AF6000; Leica, Solms, Germany). The frequency of S-phase cells was calculated as the ratio of BrdU-positive nuclei to total DAPI-stained nuclei.

### *In situ* hybridization

The plasmid for generating digoxigenin (DIG)-labeled *Bsp* RNA probes was provided by Prof. David Rice (Helsinki University) [22]. *In situ* hybridization on tissue sections was performed using *In situ* Hybridization Reagents (316-01951; Nippon Gene) according to the manufacturer's instructions. Acetylation was then carried out for 15 min in acetylation buffer (0.1 M triethanol amine-HCl, pH 8.0) containing 0.5% acetic anhydride. Next, prehybridization was performed in 50% formamide in 2× SSC for 30 min at 42°C. Each riboprobe was diluted to a final concentration of 1 µg/mL in hybridization buffer (50% formamide, 2× SSC, 1 µg/mL torula yeast RNA, 1 µg/mL salmon sperm DNA, 1× Denhardt's solution, and 10% dextran sulfate) and hybridized with sections at 42°C for 16 h. After hybridization, excess and unreacted riboprobes were removed by washing. Sections were then equilibrated for 5 min in Buffer 1 (100 mM Tris-HCl [pH 7.5] and 150 mM NaCl) and subsequently incubated in blocking buffer (1% blocking reagent [Roche Diagnostics GmbH] in Buffer 1) at room temperature for 30 min. Hybridized DIG-riboprobes were detected using anti-DIG antibodies conjugated with alkaline phosphatase (Roche Diagnostics GmbH) diluted in blocking buffer (1∶500) at room temperature for 2 h. After washes, sections were equilibrated with Buffer 3 (100 mM Tris-HCl [pH 9.0], 100 mM NaCl, and 50 mM MgCl_2_) for 15 min. Signals for each section were detected by incubation with nitroblue tetrazolium and 5-bromo-4-chloro-3-indolyl phosphate solution (Roche Diagnostics GmbH) diluted in Buffer 3 at room temperature until the optimal signal/background ratio was achieved. Color reactions were stopped by washing sections with Buffer 4 (10 mM Tris-HCl [pH 8.0] and 1 mM EDTA) for 5 min, followed by a final wash with PBS. All sections were then mounted with 90% glycerol.

### Statistical analysis

Analysis of variance (ANOVA) and the Student-Newman-Keuls test were used to analyze the results of proliferation assays. The Mann-Whitney U-test with Bonferroni correction was used to analyze the results of real-time PCR. *P* values of less than 0.01 were considered significant.

## Results

### Calvarial coronal suture cells of AS mice showed enhanced osteoblastic differentiation and increased *Fgfr2IIIb*, Fgf10, and Esrp1 expression

**Figure 1:**
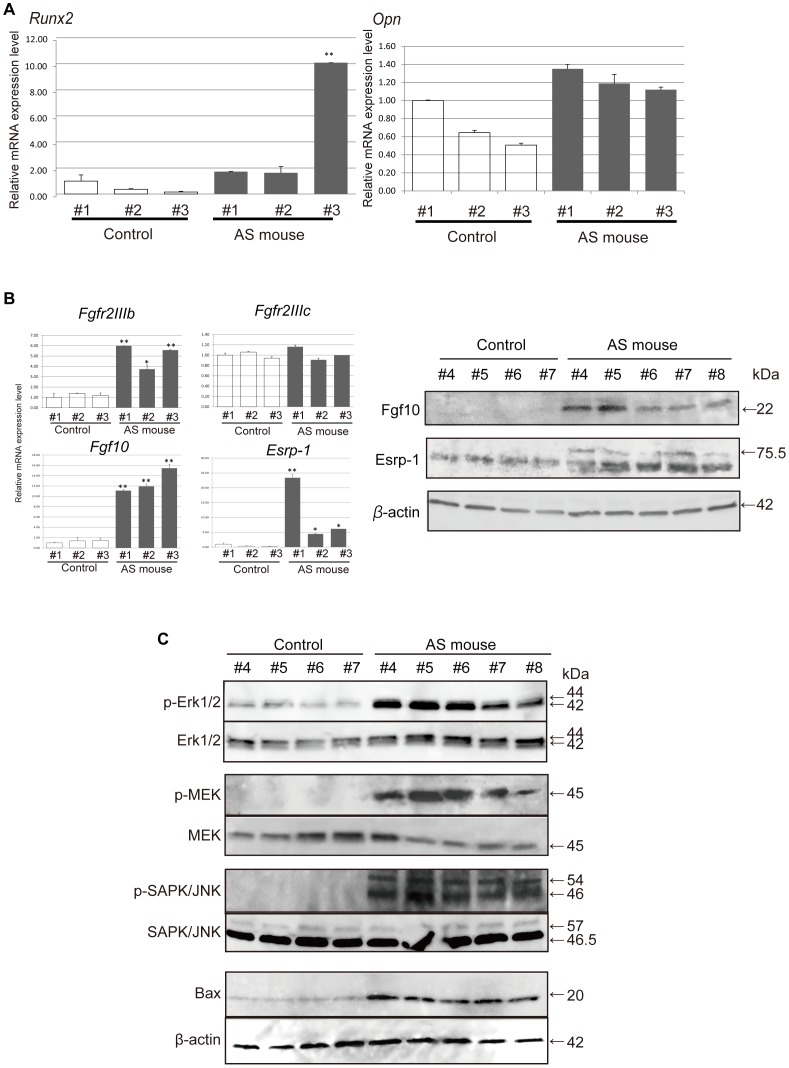
Phenotypes of calvarial coronal sutures isolated from Apert syndrome (AS) mice. (A) Embryonic calvarial coronal sutures (E15.5) were dissected from AS mice and control littermates. The mRNA expression of Runt-related transcription factor 2 *(Runx2)* and Osteopontin in calvarial coronal sutures of AS mice and littermate controls were determined by real-time PCR. Expression levels are shown as the mean ± SEM of three replicates, relative to a control #1 for each gene. (B) The mRNA expression levels of fibroblast growth factor receptor 2 (*Fgfr2*) *IIIb* and *IIIc* isoforms, *Fgf10*, and *Esrp1* were determined by real-time PCR. Increased expression of *Fgfr2IIIb, Fgf10, and Espr1* mRNA was observed in AS mice, whereas *Fgfr2IIIc* was expressed equally in both AS and control mice. The protein expression of Fgf10 and epithelial splicing regulatory protein 1 (Esrp1) in calvarial coronal sutures of AS mice and littermate controls was determined by western blot analysis. Increased expression of Fgf10 and Esrp1 was observed only in AS mice. *p<0.01, **p<0.005. (C) Phosphorylation of intracellular signaling molecules in calvarial coronal sutures was determined by western blot analysis. In AS mice, phosphorylation of Erk1/2, MEK, and SAPK/JNK were significantly enhanced, and the expression of Bax was increased.

Previous reports have shown that the coronal sutures of AS mice display premature fusion at approximately E18.5 [23], [24], [25]. To elucidate the mechanisms underlying the premature closure of suture tissue, we first investigated the expression of osteoblastic marker genes in the embryonic calvarial coronal suture of AS mice at E15.5. Real-time PCR analysis showed higher levels of Runt-related transcription factor 2 (*Runx2*) and Osteopontin (*Opn*) mRNA (Figure 1A) in the calvarial coronal sutures of AS mice compared to that in control mice. Interestingly, we observed significantly increased mRNA expression of *Fgfr2IIIb*, the epithelial splicing form of *Fgfr2*, in the coronal sutures of AS mice compared with those of littermate control mice. In contrast, uniform *Fgfr2IIIc* mRNA expression was observed in the tissues of both AS and control mice (Figure 1B). The expression of epithelial splicing regulatory protein 1 (Esrp1), which plays an important role in the regulation of *FGFR2IIIb* expression, was detected only in AS mice (Figure 1B). In addition, the Fgf10 protein, a ligand of FGFR2IIIb, was also exclusively observed in the suture tissue of AS mice (Figure 1B). From our western blot analysis, we found increased phosphorylation of intracellular signaling molecules, such as ERK1/2, MEK, and SAPK/JNK, and higher expression of Bax in the coronal sutures of AS mice, compared to those of littermate controls (Figure 1C). Therefore, these data suggested that the coronal sutures of AS mice exhibited enhanced phosphorylation of MAPK signaling molecules following activation of FGF signaling and induction of apoptosis. Taken together, these results not only confirmed that osteoblastic differentiation was strongly enhanced in the coronal suture tissues of AS mice but also suggested that the S252W mutation in the *FGFR2* gene may activate both mesenchymal and epithelial signal transduction pathways through FGF-FGFR in these tissues.

### sFGFR2IIIc^S252W^ binds to Fgf2

**Figure 2:**
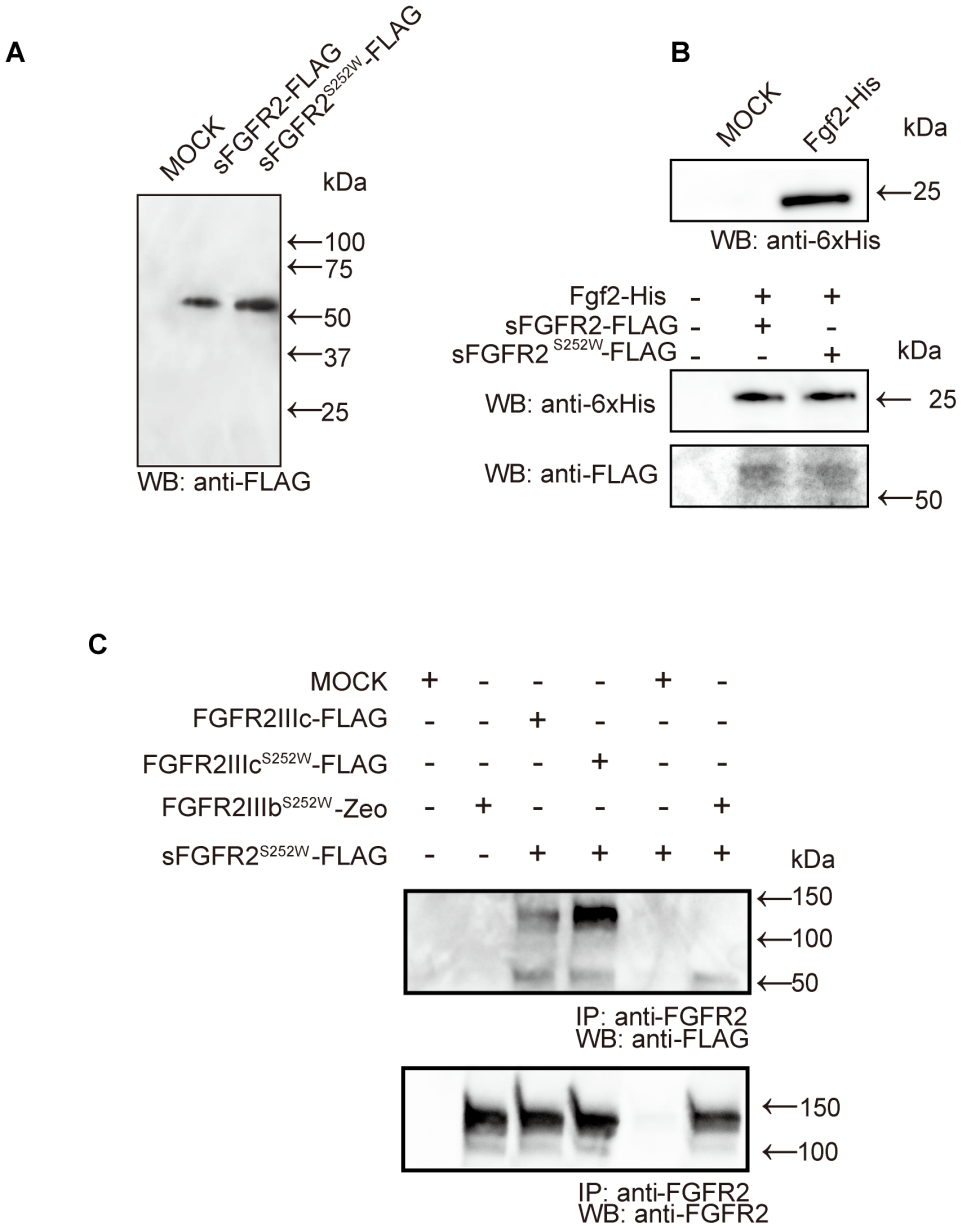
Purification of soluble forms of FGFR2 and their biological activities *in vitro*. (A, B) The expression levels of purified sFGFR2IIIc and sFGFR2IIIc^S252W^ proteins (A), as well as that of Fgf2-His (B), were analyzed by western blot analysis. (B) Pull-down assay for the binding of Fgf2-His with sFGFR2IIIc-FLAG and sFGFR2IIIc^S252W^-FLAG. Fgf2 interacted with sFGFR2IIIc-FLAG and sFGFR2IIIc^S252W^-FLAG. (C) Dimerization between sFGFR2IIIc^S252W^ and FGFR2IIIc, FGFR2IIIc^S252W^, or FGFR2IIIb^S252W^ was determined by immunoprecipitation assays using anti-FGFR2 antibodies that recognized the cytoplasmic region of Fgfr2, followed by western blot analysis using anti-FLAG antibodies. The formation of heterodimers between sFGFR2IIIc^S252W^-FLAG and membrane-bound FGFR2IIIc-FLAG, FGFR2IIIc-FLAG, FGFR2IIIc^S252W^-FLAG, or FGFR2IIIb^S252W^-FLAG was confirmed.

To identify which ligands bound to sFGFR2IIIc^S252W^ and verify whether the corresponding receptors dimerized with sFGFR2IIIc^S252W^, sFGFR2IIIc^S252W^ and the soluble form of wild-type FGFR2 (sFGFR2IIIc) were purified. A single band of approximately 50 kDa was observed in each protein preparation in the western blot analysis (Figure 2A). In addition, we also generated Fgf2-His proteins as ligands for FGFR2IIIc (Figure 2B). The results of the pull-down assays showed that both purified sFGFR2IIIc and sFGFR2IIIc^S252W^ bound to Fgf2 (Figure 2B). Next, we examined whether sFGFR2IIIc^S252W^ formed heterodimers with membrane-bound or cytoplasmic FGFR2 to abolish downstream signaling. Incomplete assembly of the intracellular signaling complex formed by dimerization of sFGFR2IIIc^S252W^ on the cell membrane or cytoplasmic FGFR2 would in turn lead to inhibition of subsequent signaling because of the formation of incomplete dimers. Since both FGFR2IIIc and FGFR2IIIb isoforms were present in the calvarial coronal sutures of AS mice (Figure 1B), we hypothesized that sFGFR2IIIc^S252W^ may dimerize with both isoforms and exert inhibitory effects on the activation of FGF signaling as a decoy receptor due to the loss of ligand specificity. To confirm this hypothesis, double transfection of Cos-7 cells with various plasmid pairs of FGFRs and ligands was performed, followed by immunoprecipitation assays (Figure 2C). Our results clearly demonstrated that sFGFR2IIIc^S252W^ formed heterodimers with FGFR2IIIc, FGFR2IIIc^S252W^, and FGFR2IIIb^S252W^ (Figure 2C).

### sFGFR2IIIc^S252W^ inhibited FGF2-stimulated phosphorylation of intracellular signaling and mineralization of MC3T3-E1 cells overexpressing FGFR2IIIc^S252W^ (MC3T3-Ap cells)

**Figure 3:**
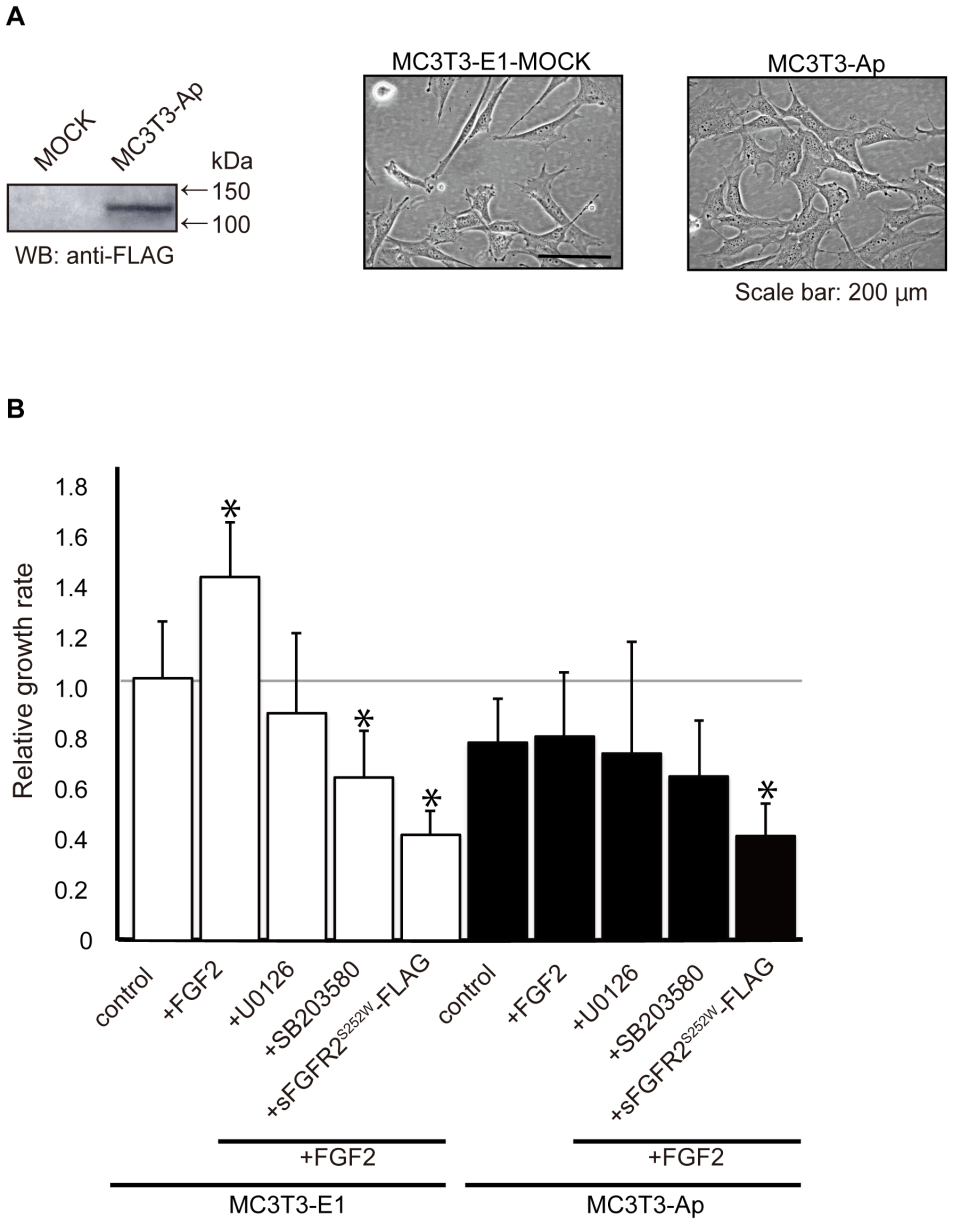
Analysis of the inhibitory effects of sFGFR2IIIc^S252W^ on the proliferation of MC3T3-E1 cells. (A) Protein expression of FGFR2IIIc^S252W^-FLAG in the established MC3T3-E1 cell clone (MC3T3-Ap) was confirmed by western blot analysis using anti-FLAG antibodies. Morphological examination of MC3T3-E1-MOCK (left) and MC3T3-Ap (right) indicated that MC3T3-Ap had a somewhat cuboidal shape, unlike the controls. (B) MTT assays were used to measure the proliferation of parental MC3T3-E1 and MC3T3-Ap cells upon administration of FGF2 (25 ng/mL) in the presence of U0126 (20 µM), SB203580 (20 µM), or sFGFR2IIIc^S252W^ (60 ng/mL). FGF2 was found to promote the proliferation of parental MC3T3-E1 cells, but not MC3T3-Ap cells. Basal proliferation levels of MC3T3-Ap cells were lower than those of parental MC3T3-E1 cells. Additionally, U0126 and SB203580 (ERK and p38 inhibitors, respectively) inhibited the proliferation of MC3T3-E1 cells. The proliferation of all cell lines was significantly decreased by addition of sFGFR2IIIc^S252W^. Statistical analysis was performed using ANOVA and the Student-Newman-Keuls test. *p<0.01.

**Figure 4:**
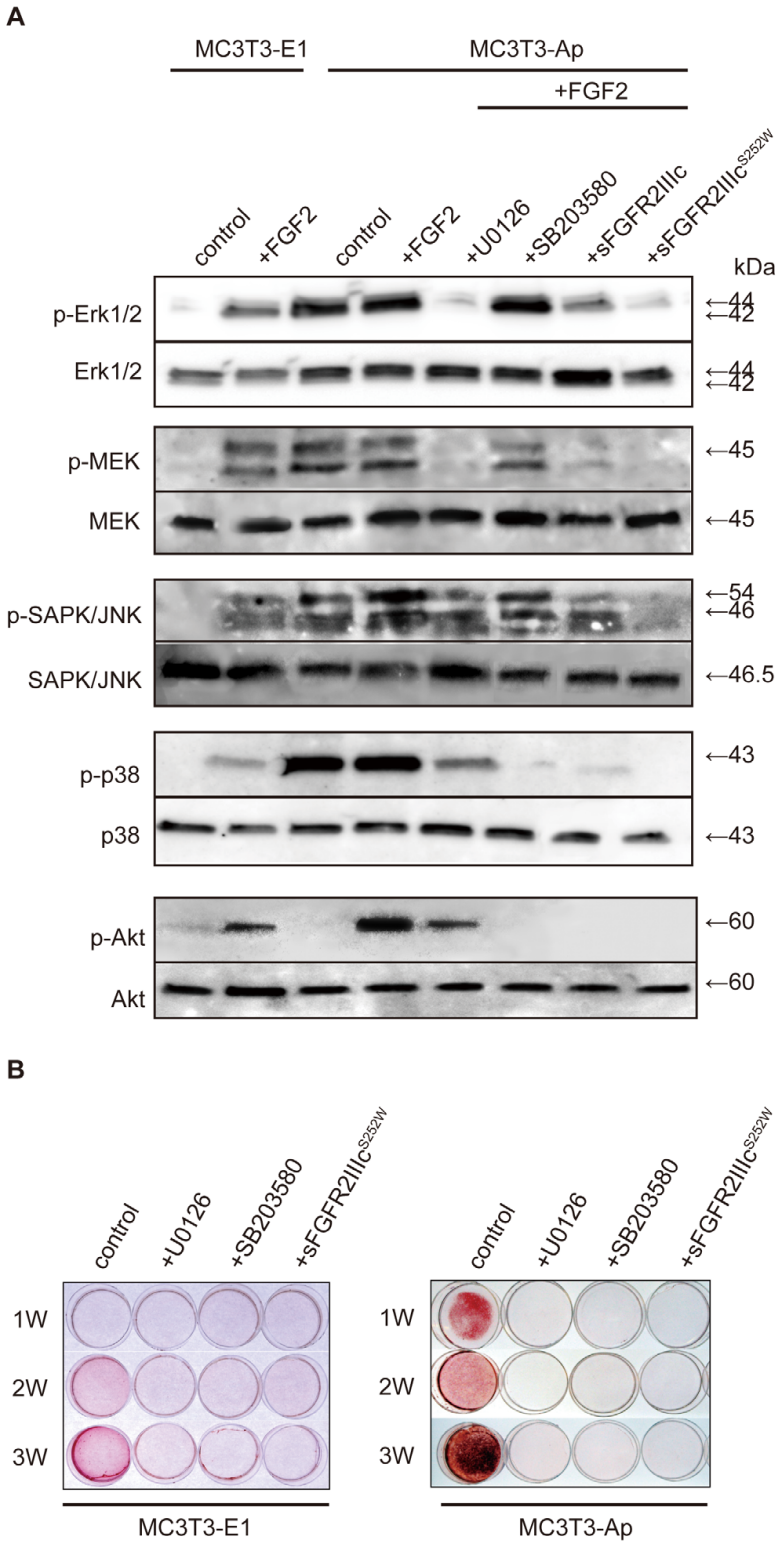
Analysis of the inhibitory effects of sFGFR2IIIc^S252W^ on intracellular signaling and *in vitro* mineralization. (A) Phosphorylation of Erk1/2, p38, MEK, SAPK/JNK, and Akt in parental MC3T3-E1 or MC3T3-Ap cells upon addition of FGF and sFGFR2IIIc^S252W^ was determined by western blot analysis. FGF2 stimulated the phosphorylation of Erk1/2, MEK, SAPK/JNK, p38, and Akt in parental MC3T3-E1 cells. MC3T3-Ap cells exhibited increased phosphorylation of all proteins except Akt. U0126 was found to inhibit the phosphorylation of Erk1/2, MEK, p38, and Akt, whereas SB203580 inhibited p38 and Akt, but not Erk1/2, MEK, or SAPK/JNK. Treatment with either sFGFR2IIIc or sFGFR2IIIc^S252W^ inhibited the phosphorylation of all of these molecules; in particular, sFGFR2IIIc^S252W^ had stronger inhibitory effects than sFGFR2IIIc on Erk1/2, SAPK/JNK, and p38. (B) Mineralization of control MC3T3-E1 cells and MC3T3-Ap cells in the presence or absence of sFGFR2IIIc^S252W^, U0126, or SB203580 was visualized by Alizarin Red S staining. MC3T3-Ap cells showed enhanced mineralization at 1 week, compared to control MC3T3-E1 cells, and administration of U0126, SB203580, or sFGFR2IIIc^S252W^ inhibited mineralization.

Western blot analysis using anti-FLAG polyclonal antibodies confirmed the expression of FGFR2IIIcS252W-FLAG in MC3T3-Ap cells (Figure 3A). Morphological examination indicated that MC3T3-Ap cells had a somewhat cuboidal shape, unlike the controls (Figure 3A). Application of FGF2 promoted the proliferation of MC3T3-E1 cells, but not that of MC3T3-Ap cells (Figure 3B), most likely because of the aberrant activation of FGF signaling in mutant cells. Basal proliferation activity of MC3T3-Ap cells appeared to be lower than that of MC3T3-E1 cells, but no significant differences were found. Additionally, administration of U0126 and SB203580 (ERK and p38 inhibitors, respectively) inhibited the FGF2-stimulated proliferation of MC3T3-E1 cells. Cell proliferation was significantly decreased by adding sFGFR2IIIc^S252W^ in both cell lines (Figure 3B), suggesting that sFGFR2IIIc^S252W^ acted as a potential inhibitor for proliferation of osteoblasts with enhanced FGF signaling. Western blot analysis using antibodies against phosphorylated MAPK signaling molecules showed that addition of FGF2 stimulated the phosphorylation of Erk1/2, MEK, SAPK/JNK, p38, and Akt in MC3T3-E1 cells (Figure 4A). On the other hand, MC3T3-Ap cells exhibited spontaneous phosphorylation of all of these molecules, with the exception of Akt (Figure 4A). While U0126 was found to inhibit the phosphorylation of Erk1/2, MEK, p38, and Akt, SB203580 inhibited the phosphorylation of p38 and Akt, but not Erk1/2, MEK, or SAPK/JNK (Figure 4A). SB203580 is a specific inhibitor of p38 MAPK and also inhibits the phosphorylation of Akt when used at higher concentrations than required to inhibit p38 MAPK [26]. Thus, these data suggested that the concentration we used was sufficient to inhibit the phosphorylation of Akt. In addition, treatment with either sFGFR2IIIc or sFGFR2IIIc^S252W^ inhibited the phosphorylation of all of these molecules; in particular, sFGFR2IIIc^S252W^ had more potent inhibitory effects than sFGFR2IIIc on Erk1/2, SAPK/JNK, and p38 (Figure 4A). These results suggested that sFGFR2IIIc^S252W^ had an inhibitory effect on activated FGF signaling by universally suppressing the phosphorylation of MAPK signaling molecules. We found that mineralization of MC3T3-E1 cells occurred within 3 weeks, whereas mineralization of MC3T3-Ap cells was observed within 1 week (Figure 4B). In addition, sFGFR2IIIc^S252W^ inhibited the mineralization capacity of both cell types, similar to the effects of U0126 and SB203580. Together, these data indicated that sFGFR2IIIc^S252W^ had an inhibitory effect on differentiation and mineralization of MC3T3-E1 cells overexpressing mutated FGFR2IIIc.

### Application of sFGFR2IIIc^S252W^ with nanogel-crosslinked hydrogels maintained the patency of cranial coronal sutures in AS mice

**Figure 5:**
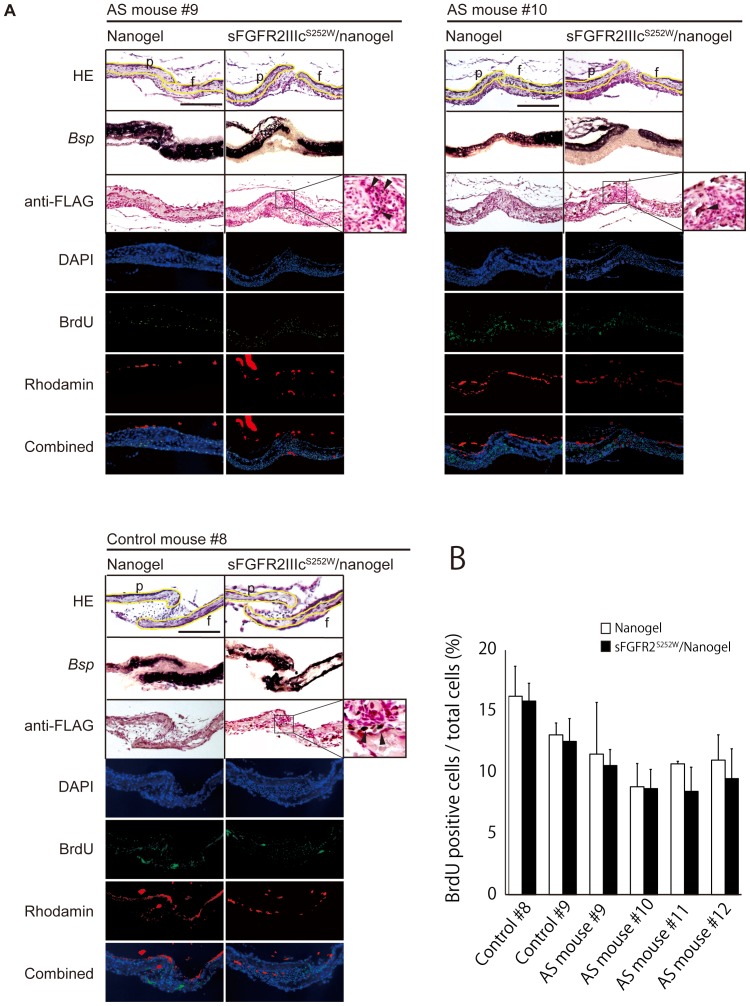
Analysis of the inhibitory effects of sFGFR2IIIc^S252W^ on the premature fusion of coronal sutures. (A) The effects of nanogel-crosslinked hydrogels incorporating sFGFR2IIIc^S252W^ on the coronal sutures of AS mice were determined using a tissue culture system. Mature bones are shown in purple, resulting from *in situ* hybridization using the bone sialoprotein (*Bsp*) probe. Yellow lines on HE-stained images show the contours of the parietal and frontal bony edges, whereas a black arrowhead shows the existence of FLAG-tagged proteins in the calvarial tissue, as determined by immunohistochemistry using anti-FLAG antibodies. The images of blue fluorescence (4′, 6-diamidino-2-phenylindole [DAPI], staining the nucleic acids), green fluorescence (bromodeoxyuridine [BrdU], incorporated into cellular DNA during proliferation), and red fluorescence (rhodamine-stained nanogels) were merged. Administration of sFGFR2IIIc^S252W^/nanogel complex maintained the patency of the coronal sutures in AS mice (n = 4/4; AS mice Nos. 9 and 10 are shown as representative examples); however, synostosis was observed on the side where only the nanogel was applied (n = 4/4). Control mice (n = 2/2; control mouse No. 8 is shown as a representative example) did not show any fusion between the frontal and parietal bones. Scale bar = 200 µm. f, frontal bone; p, parietal bone. (B) The ratios of BrdU-positive cells in AS mice and littermate controls are shown. Although no significant difference was observed, the ratio tended to be decreased in the suture tissue of AS mice, compared to the control littermates. Statistical analysis was performed using ANOVA and the Student-Newman-Keuls test.

Finally, to confirm the therapeutic applicability of sFGFR2IIIc^S252W^ for craniosynostosis, we applied a tissue culture system that used calvarial tissues dissected from AS mice and littermate controls. We performed preliminary experiments in which organs were cultured in the absence of nanogel. Using HE staining of serial sections, we confirmed that coronal sutures remained patent in control mice (n = 4/4), while AS mice exhibited synostosis of the coronal sutures (n = 4/4) after 4 days of culture in this system (Figure S1). Next, nanogel-crosslinked hydrogels complexed with or without sFGFR2IIIc^S252W^ were placed on separate sides of the coronal sutures of the calvarial bone (Figure S1). We found that nanogel-crosslinked hydrogels incorporating sFGFR2IIIc^S252W^ were able to maintain the patency of coronal sutures in AS mice (n = 4/4); however, synostosis was observed on the side where only the nanogel was applied (n = 4/4), as demonstrated by *in situ* hybridization for the bone sialoprotein (*Bsp*) gene (Figure 5A). The ratio of BrdU-positive cells to total cells tended to be lower on the side where the complex was applied even though without significant difference. Notably, in coronal sutures from AS mice (#9–#12), the ratio of BrdU-positive cells was smaller than that in the controls (#8 and #9; Figure 5B).

## Discussion

In this study, we characterized the phenotypes and FGF/FGFR signaling pathways of the coronal suture in AS mice. Application of the purified soluble form of FGFR2 carrying the S252W mutation suppressed the aberrant characteristics of osteoblasts overexpressing FGFR2IIIc^S252W^ and prevented premature fusion of the coronal suture in cultures of calvarial tissue from AS mice.

Mesenchymal tissue from coronal sutures from AS mice exhibited enhanced differentiation of osteoblasts (Fig. 1A) and increased phosphorylation of ERK, MEK, and SAPK/JNK (Figure 1C). Previously, we reported marked premature ossification of the callus during the waiting period of distraction osteogenesis for the treatment of the deformed thumb in a patient with Apert syndrome (S252W) [27]. Consistent with previous reports showing that FGFR2-dependent osteogenesis was promoted by the Apert mutation [28], [29], the clinical findings suggested that FGFR2 bearing the S252W mutation promotes osteogenic ability. We have reported the accelerated mineralization of osteoblast-like cells in two patients with Apert syndrome [8], MG63 osteosarcoma clones (n = 4) stably expressing FGFR2IIIcS252W (the Apert mutation) [8], and transgenic mice overexpressing FGFR2IIIcS252W *in vitro* and *in vivo* [9]. Miraoui et al. reported that FGFR2IIIcS252W promoted osteogenic gene expression through ERK1/2 and protein kinase C alpha (PKCα) signaling and accelerated mineralization of murine mesenchymal C3H10T1/2 [30]. Apoptosis has been proposed as the underlying mechanism of suture fusion [23]. However, Holmes et al. concluded that apoptosis is likely to be a consequence rather than a cause of synostosis even though they showed apoptotic cells are present in the limited osteoid contact in the Apert mutant coronal sutures after E16.5 [24]. Meanwhile, recent study reported a high percentage of apoptotic chondrocytes in Axin2 (−/−) posterior-frontal suture which showed significant closure delay at the early postnatal stage [31]. As mentioned above, the relationship between apoptosis and pathological premature suture closure or closure insufficiency does not reach a definitive conclusion. In this study, we observed an increased expression of Bax in the coronal suture of AS mice at E15.5 (Figure 1C), suggesting the early incidence of apoptosis in the coronal suture of Apert mice. Given our previous studies [8], [9], we believe that the main causative mechanism is the enhanced osteoblast differentiation in the osteogenic front and sutural mesenchymal cells of the prematurely fused suture. This phenomenon could conductive to lower cell proliferation and apoptosis. Further studies are needed in order to clarify the detail role of apoptosis in the pathological suture development. We also observed increased expression of *Esrp1*, *FGFR2IIIb*, and *Fgf10* mRNAs in AS mouse coronal sutures (Figure 1B). Previous studies have shown that ESRP1 and ESRP2 are required for the expression of epithelial FGFR2IIIb [32]. The Fgfr2IIIb isoform is known to be expressed in epithelial cells and has been shown to bind to Fgf7 and/or Fgf10 with high affinity [33]. *FGFR2IIIb* expression in the maxillofacial region of AS patients has not been examined; however, fibroblast cells obtained from the limbs of AS patients carrying an *Alu*-element insertion in *FGFR2* have been reported to exhibit ectopic expression of *FGFR2IIIb* [34]. Molecular studies have shown that *Alu* insertions affect the alternative splicing of *FGFR2*, resulting in ectopic expression of FGFR2IIIb in mesenchyme-derived cell lineages that normally express FGFR2IIIc [34]. Hajihosseini and colleagues [35] reported that mice with heterozygotic abrogation of *Fgfr2*-exon 9 (IIIc) exhibit craniosynostosis and that *Fgfr2IIIb* is strongly expressed in calvarial sutures. The authors hypothesized that there is less Fgfr2IIIc because of the Cre deletion in these calvarial sutures, leading to increased *FGFR2IIIb* expression. The S252W or P253R mutation in FGFR2 allows FGFR2IIIc to be activated by FGF7 or FGF10 [6]. Based on these findings, we suggest that increased Fgfr2IIIb/Fgf10 expression contributes to the onset of craniosynostosis in AS mice. Rice et al. reported that the expression of *Fgfr2IIIb* mRNA was detected by *in situ* hybridization in the cranial base perichondrium and periosteum at very low levels [36]. In addition, *in situ* hybridization revealed minute amounts of *Fgfr2IIIb* mRNA at the osteogenic front of the sagittal suture at E15 and E17 [37] and at the developing primordia of the frontal bone [38]. Veistinen et al. showed low expression of *Fgf10* mRNA in the developing primordia of the frontal bone by *in situ* hybridization [38]. These results prompted us to perform real-time PCR to assess the expression levels of *Fgfr2IIIb*, *Fgf10*, and *Esrp1* in the coronal sutures of our AS mice (E15.5). We detected the expression of *Fgfr2IIIb*, *Fgf10*, and *Esrp1* mRNA by real-time PCR; however, Fgf10 and Esrp1 proteins were not detected in control mice in our western blot analysis. Because the expression levels of these genes at the wild-type calvarial suture were low, the corresponding protein levels might have been too low to detect under our experimental conditions. Further analysis will be required to investigate the expression and the function of FGFR2IIIb, particularly with respect to calvarial bone development in human craniosynostosis. We observed a downward trend in the number of BrdU-positive cells in Apert calvarial tissues; however, this difference was not significant (Figure 5). This phenomenon might reflect the aberrantly enhanced MAPK signaling in Apert calvaria, as shown in Figure 1C. Previously, we reported a reduction of proliferation rate in MG63 osteosarcoma cells overexpressing FGFR2IIIcS252W [8]. Calvarial tissues from Apert mice reportedly formed less bone than wild-type mice during a 14-day calcein labeling period, and [^3^H]-thymidine incorporation indicated a slight decrease in cell proliferation in mutant coronal sutures [23]. Although the mechanisms responsible for these phenomena has not been identified, recent studies have shown extensive crosstalk between canonical Wnt/β-catenin and MAPK signaling in melanoma cells [39]. Hyperactivated MAPK signaling has been shown to stabilize the tumor suppressor protein Axin inhibitor, which inhibits Wnt signaling in melanoma. Bax-induced apoptosis has been proposed as possible mechanism of pathological premature suture fusion [23]. For example, Holmes et al. found that apoptotic cells appeared in Apert mutant coronal sutures after E16.5 [24], while Fong et al. reported that the number of apoptotic cells increased during postnatal posterior-frontal suture closure [40]. We observed an increase in Bax expression in the coronal sutures of AS mice at E15.5 (Figure 1C) suggesting that the coronal sutures of AS mice underwent apoptosis due to aberrant FGFR signaling.

Our data suggested that the purified sFGFRIIIc^S252W^ possessed a ligand-binding domain that bound to FGF2 (Figure 2B). We also demonstrated that the FGFR2IIIc^S252W^ and FGFR2IIIb^S252W^ isoforms both formed dimers with FGFRs (Figure 2C). From these results, we speculated that sFGFR2IIIc^S252W^ abrogated activated Fgf/Fgfr2 signaling through binding to extracellular Fgf ligands, and through receptor dimerization with monomeric Fgfr2 expressed on the cell membrane or within the cytoplasm. We generated the clone of MC3T3-Ap (Figure 3A) and demonstrated that the proliferation of these MC3T3-Ap cells was significantly decreased by sFGFR2IIIc^S252W^. No significant difference in proliferation was observed between intact MC3T3-E1 and MC3T3-Ap cells (Figure 3B). These data suggested that sFGFR2IIIc^S252W^ acted as an inhibitor for FGF2-stimulated proliferation. Our data also suggested that MC3T3-Ap mimicked the phenotypes of AS calvarial osteoblasts, exhibiting enhanced osteoblastic differentiation and phosphorylation of intracellular signaling molecules (Figures 1A, 1C and 4A, and 4B). These observations are consistent with those of previous studies showing that the S252W mutation increases osteoblast differentiation [7], [9], [24], [25]. The differentiation capacity of MC3T3-Ap cells was significantly increased compared to intact MC3T3-E1 cells, whereas the cells treated with sFGFR2IIIc^S252W^ showed decreased proliferation and differentiation (Figures 3B and 4B). Increased differentiation is possibly a specific effect of the S252W mutation in osteoblasts. In terms of the etiological mechanism of craniosynostosis in AS, mutation of FGFR2 at either S252W or P253R has been reported to cause loss of ligand specificity and decrease the dissociation rate of FGFs from FGFR2, resulting in activated FGF/FGFR signaling. Ligand-dependent activation of FGF/FGFR signaling induces osteoblastic differentiation, potentially leading to the hypercalcification of sutural mesenchymal tissue.

The MEK/ERK inhibitor PD98059 has been shown to partially inhibit craniosynostosis in cultures of calvarial tissues from AS mice carrying the P253R mutation [41]. RNA interference and inhibition of MEK/ERK signaling also rescued craniosynostosis in AS mice carrying the S252W mutation [42]. Specifically, shRNAs targeting to *Fgfr2^S252W^* relieved premature fusion of coronal sutures *in vivo* [42]. In addition, recombinant Noggin has been reported to act as an inhibitor of craniosynostosis in rat calvarial coronal sutures with transplantation of human AS osteoblasts [43]. However, these strategies are currently distant from clinical applications. Therefore, because surgical intervention is the only current treatment available to reduce morphological and function defects in patients with AS, noninvasive procedures for treatment of AS need to be developed. To this end, our group has focused on the soluble form of FGFR2 and its dominant-negative effect on FGFs [8], [9], [10]. We found that sFGFR2IIIc^S252W^ was able to maintain the patency of the AS mouse coronal sutures in the calvarial tissue culture system (Figure 5A). Some basic studies have examined the proficiency of soluble receptors to function as decoy receptors in clinical applications [44], [45]. Our purified sFGFR2IIIc^S252W^ is unique for several reasons. First, we speculate that soluble FGFR2IIIc^S252W^ can spread to the whole body through the bloodstream because of its endogenous expression in human tissues. Second, it has potent inhibitory effects towards the pathological conditions of AS because of the S252W mutation, which causes loss of ligand specificity and reduces ligand dissociation rates; this study is the first to report the use of a soluble receptor carrying a mutation associated with a human congenital anomaly. Third, purified sFGFR2IIIc^S252W^ not only has binding affinity for Fgf2, but was also shown to dimerize with membrane-bound or cytoplasmic monomeric FGFR2 in the presence or absence of the S252W mutation (Figure 2C), resulting in incomplete dimers that block subsequent intercellular signaling. Our current strategy, which applied the purified protein to the calvarial tissue by using a nanogel-crosslinked hydrogel system as the protein carrier has several advantages; individual nanogels can store proteins inside their nano spaces and then gradually release proteins locally without any significant change in their biological activities; they do not contain antigens; they are inexpensive. However, many problems must be resolved before this protein is applied in clinical settings for the treatment of AS patients. Because craniosynostosis typically occurs during fetal life [46], the safety and effectiveness of FGFR2-based treatment must be tested and an appropriate administration technique should be established using animal experiments *in vivo*. Some patients with craniosynostosis display recrudescence of bony fusion just after craniectomy; the complex might help block these unfavorable phenomena caused by aberrant FGF/FGFR signaling. Taken together, our data suggested that the appropriate delivery of purified sFGFR2IIIc^S252W^ could be an effective method for treating not only AS but also other types of craniosynostosis resulting from aberrant FGF/FGFR signaling.

## Supporting Information

Figure S1 **Diagram of the calvarial tissue culture system in this study and reproducibility of the premature fusion of coronal sutures in AS mice.** (A) Calvarial bones (E15.5) were dissected from underlying mouse brains, and the skin was peeled off. The calvarial bone unit from each embryo was divided into two pieces along the midline interfrontal and sagittal sutures, and sectioned bones were placed in two separate dishes. The explants were then placed on filters supported by a metal mesh, with the brain side oriented down and the skin side oriented up. Explants were cultured in 10% FBS-DMEM containing 100 µg/mL ascorbic acid at 37°C in an atmosphere containing 5% CO_2_ under humidified conditions for 4 days in the presence of either nanogel-crosslinked hydrogels complexed with sFGFR2IIIc^S252W^ or vehicle nanogels placed across the coronal suture. (B, C) We performed preliminary experiments to assess the incidence of premature fusion of the coronal sutures in AS mice during the 4-days tissue culture period in the absence of nanogel. Using HE staining of serial sections, we confirmed that coronal sutures remained patent in control mice (n = 4/4), while AS mice exhibit synostosis of the coronal sutures (n = 4/4) in this system. Scale bar = 100 µm. cs, coronal suture; f, frontal bone; of, osteogenic front; p, parietal bone.
(TIF)
