# Supplementary figures and images for: Therapeutic Effect of Nanogel-Based Delivery of Soluble FGFR2 with S252W Mutation on Craniosynostosis
Source: PLoS One. 2014 Jul 8;9(7):e101693. doi: 10.1371/journal.pone.0101693 (PMC4086955; doi:10.1371/journal.pone.0101693)

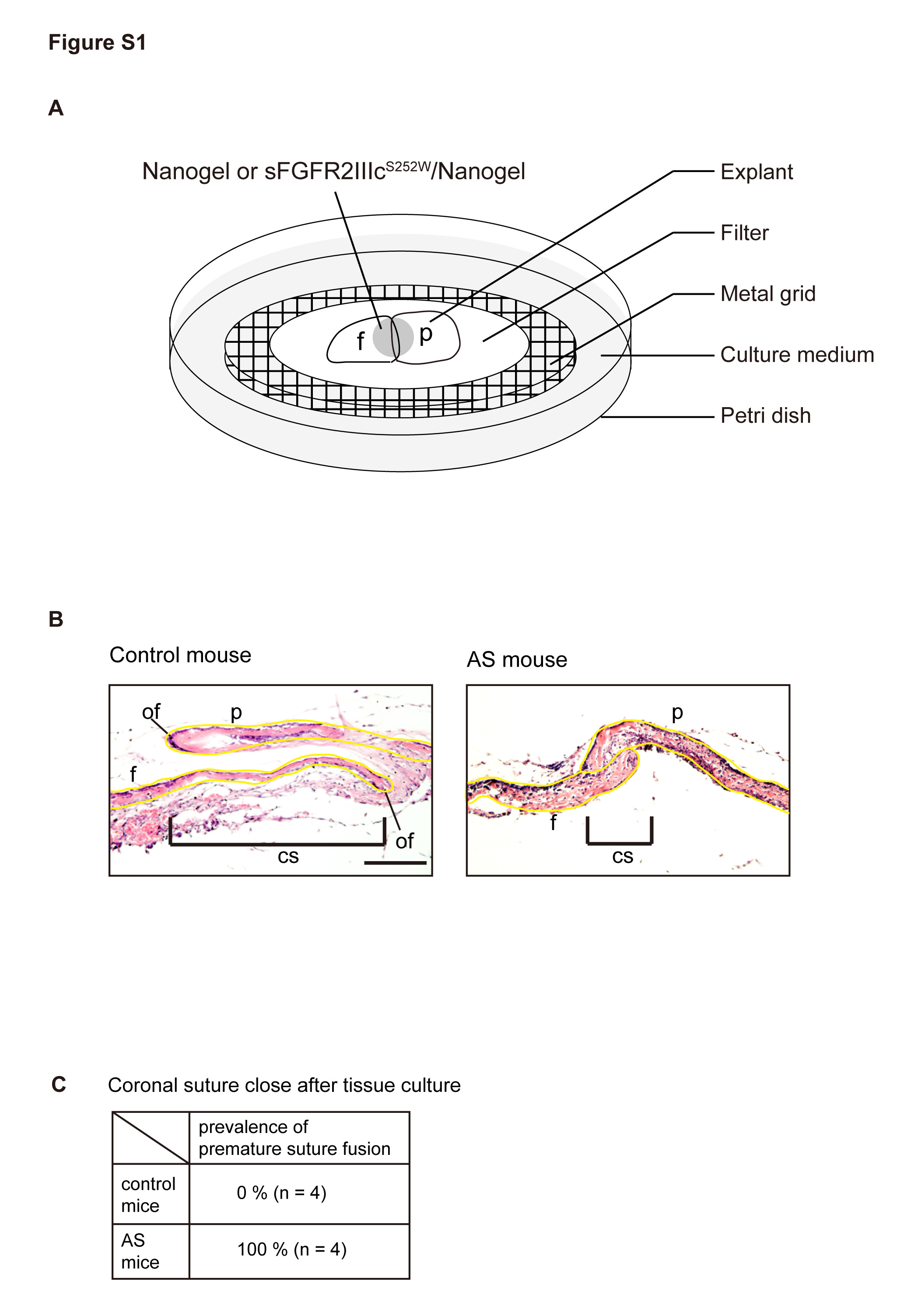

Supplement: Figure S1 — Diagram of the calvarial tissue culture system in this study and reproducibility of the premature fusion of coronal sutures in AS mice. (A) Calvarial bones (E15.5) were dissected from underlying mouse brains, and the skin was peeled off. The calvarial bone unit from each embryo was divided into two pieces along the midline interfrontal and sagittal sutures, and sectioned bones were placed in two separate dishes. The explants were then placed on filters supported by a metal mesh, with the brain side oriented down and the skin side oriented up. Explants were cultured in 10% FBS-DMEM containing 100 µg/mL ascorbic acid at 37°C in an atmosphere containing 5% CO2 under humidified conditions for 4 days in the presence of either nanogel-crosslinked hydrogels complexed with sFGFR2IIIcS252W or vehicle nanogels placed across the coronal suture. (B, C) We performed preliminary experiments to assess the incidence of premature fusion of the coronal sutures in AS mice during the 4-days tissue culture period in the absence of nanogel. Using HE staining of serial sections, we confirmed that coronal sutures remained patent in control mice (n = 4/4), while AS mice exhibit synostosis of the coronal sutures (n = 4/4) in this system. Scale bar = 100 µm. cs, coronal suture; f, frontal bone; of, osteogenic front; p, parietal bone. (TIF) [file pone.0101693.s001.tif]
